# MicroRNA-24 Regulates Osteogenic Differentiation via Targeting T-Cell Factor-1

**DOI:** 10.3390/ijms160511699

**Published:** 2015-05-21

**Authors:** Weigong Zhao, Caijun Wu, Yanying Dong, Yunfeng Ma, Yaofeng Jin, Yanhong Ji

**Affiliations:** 1 Department of Orthopedics, the First Affiliated Hospital of Medical College, Xi’an Jiaotong University, Xi’an 710061, China; 2 Department of Immunology and Pathogenic Biology, School of Basic Medical Sciences, Xi’an Jiaotong University Health Science Center, Xi’an 710061, China; E-Mails: caijunwucc@163.com (C.W.); yanyingdong11@163.com (Y.D.); yunfengma126@163.com (Y.M.); 3 Department of Pathology, the Second Affiliated Hospital of Medical College, Xi’an Jiaotong University, Xi’an 710004, China; E-Mail: yaofengjin@126.com

**Keywords:** miR-24, differentiation, osteoblast, T-cell factor-1

## Abstract

MicroRNAs (miRNAs) have been reported to have diverse biological roles in regulating many biological processes, including osteogenic differentiation. In the present study, we identified that miR-24 was a critical regulator during osteogenic differentiation. We found that overexpression of miR-24 significantly inhibited osteogenic differentiation, which decreased alkaline phosphatase activity, matrix mineralization and the expression of osteogenic differentiation markers. In contrast, inhibition of miR-24 exhibited an opposite effect. Furthermore, we delineated that miR-24 regulates post-transcriptionals of T-cell factor-1 (Tcf-1) via targeting the 3'-untranslated region (UTR) of Tcf-1 mRNA. MiR-24 was further found to regulate the protein expression of Tcf-1 in the murine osteoprogenitors cells and bone mesenchymal stem cells. Additionally, the positive effect of miR-24 suppression on osteoblast differentiation was apparently abrogated by Tcf-1 silencing. Taken together, our data suggest that miR-24 participates in osteogenic differentiation by targeting and regulating Tcf-1 expression in osteoblastic cells.

## 1. Introduction

Osteoblasts and osteoclasts play an important role in regulating bone homeostasis [1]. A reduction in bone mass results in osteoporosis and current treatments generally target osteoclasts to inhibit bone resorption [2]. However, their effects are rarely effective on bone mass recovery [3]. Therefore, therapeutic agents targeting new bone formation such as osteogenic differentiation to enhance bone building are of great importance. Osteoclasts originate from mesenchymal stem cells and are the main bone-forming cells in bone tissues. These cells are pivotal for bone formation as they generate alkaline phosphatase (ALP) and bone matrix proteins including collagen type Iα1 (ColA1) and osteopontin (OPN) to induce osteoblastic mineralization [4]. Osteoblast differentiation from osteoblast precursors is mediated by a variety of extracellular ligands including fibroblast growth factors, bone morphogenetic proteins, and Wnts and their activated signaling pathways [5].

MicroRNAs (miRNA) are small non-coding RNAs (~22 nucleotides in length) that have been reported to play critical roles in various cellular processes and diseases [6,7]. MiRNAs regulate gene expression through targeting the 3'-untranslated region (UTR) of messenger RNA (mRNA), resulting in mRNA destabilization and translation inhibition [8,9]. Differentially expressed miRNAs have been found during osteogenic differentiation and have been suggested to play an important role in osteogenic differentiation [10,11,12,13]. Mizuno *et al.* have reported that miR-210 increases osteogenic differentiation through the inhibition of activin A receptor type 1B [14]. MiR-96 has been found to promote osteogenic differentiation by inhibiting heparin-binding epidermal growth factor-like growth factor [15]. MiR-302a has been demonstrated to stimulate osteogenic differentiation via targeting and inhibiting chicken ovalbumin upstream promoter transcription factor I which is a potent transcription factor inhibiting osteogenic differentiation [16]. These findings provide evidence that targeting miRNA may provide potential and valid therapeutics for the treatment of bone mass loss.

The Wnt signaling pathway plays an important role in regulating cell apoptosis, cell growth and differentiation [17]. Wnt proteins bind with frizzled receptors and inhibits a downstream phosphorylation cascade to stabilize intracellular β-catenin levels, leading to β-catenin accumulation and translocation to the nucleus which subsequently activates gene expression of DNA-binding HMG box transcription factors and lymphoid enhancer factor/T-cell factor [18,19]. The Wnt signaling pathway has been implicated in regulating bone formation and bone mass [20,21]. T-cell factor-1 (Tcf-1) expression has been found to be highly increased in secreted frizzled-related protein-1-null mice with activated Wnt signaling and increased runt-related transcription factor 2 (Runx2) activity [22], the key osteogenic transcription factor for osteogenic differentiation and bone formation [23].

Increasing study has suggested that miR-24 is extensively involved in regulating cell differentiation such as epidermal differentiation [24], blood cell differentiation [25], and skeletal muscle differentiation [26]. However, the role and the underlying mechanism of miR-24 in regulating osteogenic differentiation remain poorly understood. In the present study, we explored the expression profile of miR-24 during osteogenic differentiation and investigated the effect of ectopic expression of miR-24 on osteogenic differentiation. Tcf-1 as a putative target gene of miR-24 was screened by bioinformatics analysis and validated by a dual luciferase reporter assay. We demonstrate here that miR-24 regulates osteogenic differentiation through targeting and regulating the expression of Tcf-1.

## 2. Results

### 2.1. miR-24 Is Involved in Osteogenic Differentiation

**Figure 1:**
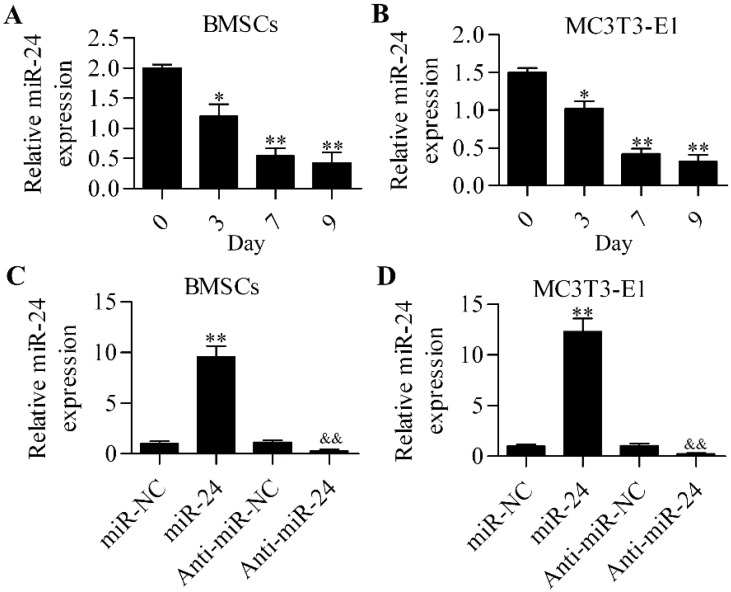
Detection of the expression of miR-24. RT-qPCR was performed to detect the expression level of miR-24 during osteogenic differentiation in BMSCs (**A**) and MC3T3-E1 cells (**B**). *N* = 3, * *p* < 0.05 ** *p* < 0.01 *vs.* day 0. RT-qPCR analysis of miR-24 expression in miR-24 or anti-miR-24 transfected BMSCs (**C**) and MC3T3-E1cells (**D**). Cells were transfected with 20 nM miR-24 or anti-miR-24 for 48 h. miR-NC or anti-miR-NC was used as the control for miR-24 or anti-miR-24, respectively. *N* = 3, ** *p* < 0.01 *vs.* miR-NC; and ^&&^ *p* < 0.01 *vs.* anti-miR-NC.

**Figure 2:**
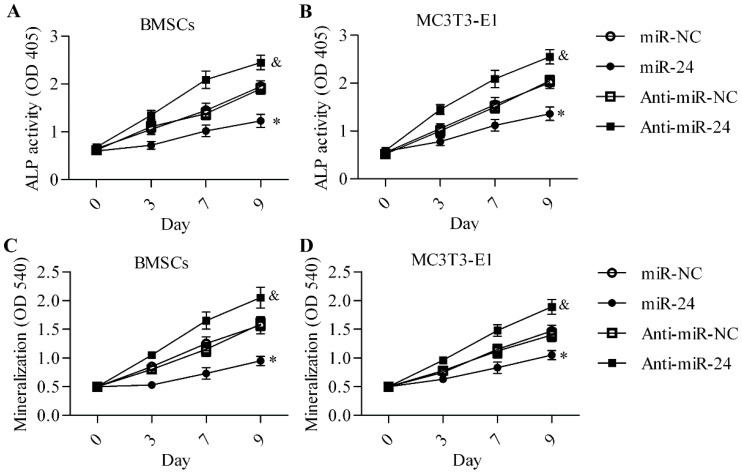
Effect of miR-24 gain or loss on osteogenic differentiation. ALP activity was detected in BMSCs (**A**) and MC3T3-E1cells (**B**) using a commercial ALP activity assay kit. Cells were transfected with miR-24 or anti-miR-24 (day 0) for 48 h and then cultured in differentiation medium for the induction of osteogenic differentiation. The absorbance at 405 nm was measured at days 3, 7 and 9; Mineralization in BMSCs (**C**) and MC3T3-E1cells (**D**) was assessed using Alizarin red S staining. The absorbance at 540 nm was quantified. *N* = 6, * *p* < 0.05 *vs.* miR-NC; ^&^ *p* < 0.05 *vs.* anti-miR-NC.

To gain insight into miR-24 in osteogenic differentiation, we first detected the expression profile of miR-24 during osteogenic differentiation by RT-qPCR. The results show that miR-24 was downregulated during osteogenic differentiation in mouse bone mesenchymal stem cells (BMSCs) (Figure 1A) and mouse embryo osteoblast precursor (MC3T3-E1) cells (Figure 1B). The data imply a potential role of miR-24 in osteogenic differentiation. To further investigate the role of miR-24 in osteogenic differentiation, we performed miR-24 gain- or loss-of-function experiments in BMSCs (Figure 1C) and MC3T3-E1 cells (Figure 1D). The overexpression of miR-24 significantly decreased alkaline phosphatase (ALP) activity, whereas downregulation of miR-24 markedly enhanced ALP activity in BMSCs (Figure 2A) and MC3T3-E1 cells (Figure 2B) during osteogenic differentiation. Furthermore, matrix mineralization in BMSCs (Figure 2C) and MC3T3-E1 cells (Figure 2D) was also downregulated or increased by miR-24 overexpression or downregulation, respectively.

### 2.2. Inhibition of miR-24 Elevates the Expression of Osteogenic Differentiation Markers

**Figure 3:**
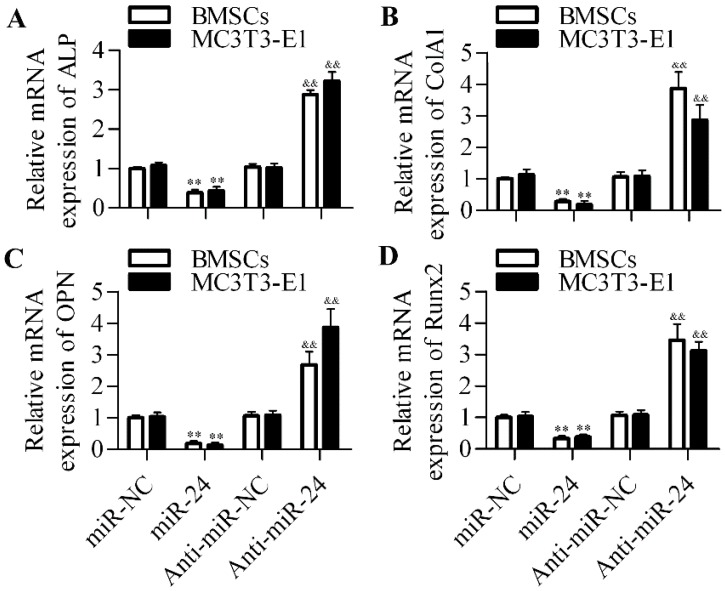
Effect of miR-24 gain or loss on the mRNA expression of osteogenesis-related genes. RT-qPCR was performed to detect the mRNA expression level of ALP (**A**), ColA1 (**B**), OPN (**C**) and Runx2 (**D**) in BMSCs and MC3T3-E1cells. Cells transfected with miR-24 or anti-miR-24 (day 0) for 48 h and then cultured in differentiation medium for induction of osteogenic differentiation. Cells were harvested at day 7 for analysis. *N* = 3, ** *p* < 0.01 *vs.* miR-NC; and ^&&^ *p* < 0.01 *vs.* anti-miR-NC.

ALP, collagen type Iα1 (ColA1), and osteopontin (OPN) have been suggested as the molecular markers of osteogenic differentiation [27]. The expression of all these genes was significantly downregulated with miR-24 overexpression, whereas inhibition of miR-24 markedly elevated the expression of all these genes (Figure 3A–C). Furthermore, the key osteogenic transcription factor Runx2 was also remarkably downregulated by miR-24 overexpression or upregulated by miR-24 inhibition (Figure 3D).

### 2.3. miR-24 Directly Targets the 3′-UTR of Tcf-1

**Figure 4:**
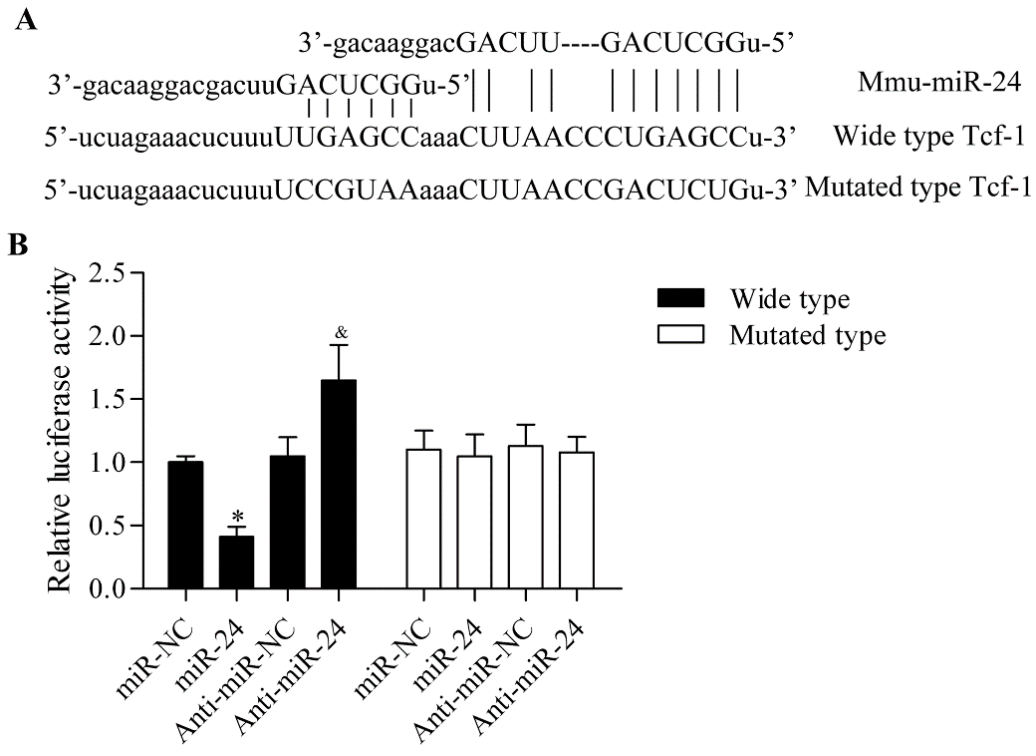
miR-24 directly targets the 3'-UTR of Tcf-1. (**A**) The 3'-UTR of Tcf-1 had the putative binding sites with miR-24; (**B**) The direct binding relationship between 3'-UTR of Tcf-1 and miR-24 was detected by a dual luciferase activity assay. The wild type or mutated 3'-UTR of Tcf-1containing the putative binding sites for miR-24 in pGL3 luciferase reporters was transfected into MC3T3-E1cells cells with miR-24 or anti-miR-24. After 48 h of incubation, cells were harvested and luciferase activity was measured by the dual luciferase reporter assay system. *N* = 6, * *p* < 0.05 *vs.* miR-NC; and ^&^ *p* < 0.05 *vs.* anti-miR-NC.

To further delineate the underlying molecular mechanism of miR-24 in regulating osteogenic differentiation, we predicted the putative target genes of miR-24 through bioinformatics analysis. Intriguingly, we found that Tcf-1, a key transcription factor downstream of Wnt/β-catenin, contained the putative binding sites for miR-24 in the 3'-UTR (Figure 4A). To confirm this direct relationship between Tcf-1 and miR-24, a dual luciferase reporter assay was performed. The wild type and the mutants of 3'-UTR of Tcf-1 in the predicted binding sequences were constructed into luciferase reporters (pGL3). The results showed that administration of miR-24 significantly downregulated luciferase activity in pGL3-Tcf-13'-UTR (wild type) transfected cells, whereas administration of anti-miR-24 markedly increased the luciferase activity (Figure 4B). Conversely, ectopic expression of miR-24 had no apparent effect on luciferase activity in pGL3-Tcf-13'-UTR (mutated type) transfected cells.

### 2.4. miR-24 Regulates Tcf-1 Expression in BMSCs and MC3T3-E1 Cells

**Figure 5:**
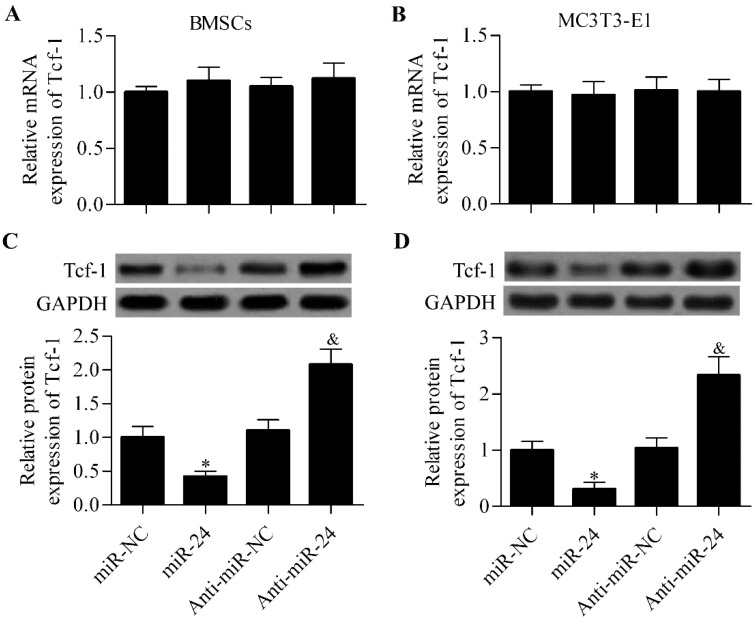
miR-24 regulates Tcf-1 expression in BMSCs and MC3T3-E1 cells. RT-qPCR analysis of Tcf-1 mRNA expression in BMSCs (**A**) and MC3T3-E1 cells (**B**) transfected with miR-24 or anti-miR-24; Western blot analysis of Tcf-1 protein expression in MSCs (**C**) and MC3T3-E1 cells (**D**). Relative protein expression level was quantified using Image-Pro Plus 6.0 software (Media Cybernetics, Inc., Rockville, MD, USA). Cells transfected with miRNAs for 48 h before harvested for analysis. *N* = 3, * *p* < 0.05 *vs.* miR-NC; and ^&^ *p* < 0.05 *vs.* anti-miR-NC.

To further validate that miR-24 targeted miR-24 and regulated the expression of Tcf-1, we investigated the effect of ectopic miR-24 expression on Tcf-1 expression in BMSCs and MC3T3-E1 cells. RT-qPCR analysis showed that the mRNA expression level of Tcf-1 was not affected by miR-24 or anti-miR-24 in BMSCs (Figure 5A) and MC3T3-E1 cells (Figure 5B). Next, we further detected their effect on the protein expression of Tcf-1 by Western blot analysis. The results show that miR-24 overexpression significantly decreased the protein expression of Tcf-1 in BMSCs and MC3T3-E1 cells (Figure 5C,D). In contrast, anti-miR-24 markedly increased the protein expression of Tcf-1 (Figure 5C,D).

### 2.5. Silencing of Tcf-1 Abolishes the Positive Effect of miR-24 Inhibition on Osteoblast Differentiation

**Figure 6:**
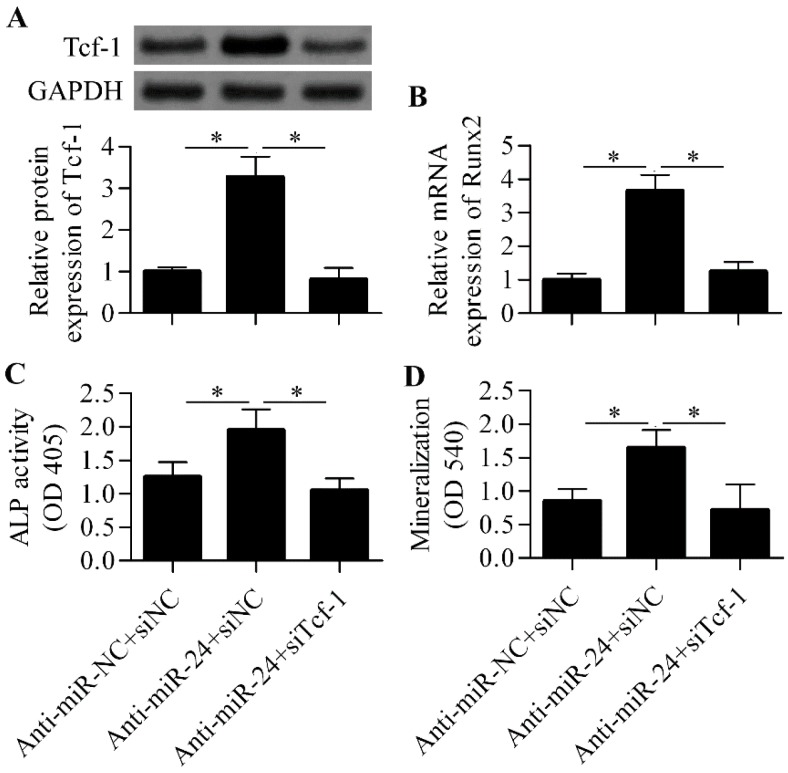
Tcf-1 knockdown abrogated the effect of anti-miR-24 on osteoblast differentiation. (**A**) Western blot analysis of Tcf-1 protein expression in anti-miR-24 transfected MC3T3-E1 cells in the presence with Tcf-1 siRNA. Anti-miR-NC and siNC were used as the control; (**B**) RT-qPCR analysis of Runx2 mRNA expression in the different groups; (**C**) ALP activity was detected by an ALP activity assay in the different groups after seven days of osteogenic differentiation; (**D**) Mineralization was assessed using Alizarin red S staining at day 7. *N* = 3, * *p* < 0.05.

To further verify that miR-24 regulated osteoblast differentiation through modulating Tcf-1 expression, we performed Tcf-1 siRNA experiments along with miR-24 inhibition. The results show that the silencing of Tcf-1 (Figure 6A) apparently abrogated the positive effect of anti-miR-24 on osteoblast differentiation in which the increased gene expression of the key osteogenic transcription factor Runx2 induced by anti-miR-24 was remarkably downregulated by Tcf-1 siRNA (Figure 6B). Furthermore, the increased ALP activity (Figure 6C) and matrix mineralization (Figure 6D) induced by anti-miR-24 were also significantly decreased by Tcf-1 siRNA.

## 3. Discussion

In recent years, the critical roles of miRNAs in osteogenic differentiation have been highlighted, indicating that miRNAs can serve as major regulators to promote or inhibit osteogenic differentiation [16,28,29,30]. In the present work, we have delineated that miR-24 is a negative regulator of osteogenic differentiation. We found that miR-24 was decreased during osteogenic differentiation and inhibition of miR-24 promoted osteogenic differentiation by increasing Tcf-1, an important target gene of the Wnt signaling pathway.

The biological roles of miR-24 have been widely investigated in many studies. MiR-24 has been reported to be overexpressed in hepatocellular carcinoma and inhibition of miR-24 significantly represses cell proliferation, invasion, and migration by targeting sex-determining region Y-box 7 [31]. However, miR-24 was also found to be a tumor suppressor gene that inhibited gastric cancer progression by decreasing the gene expression of regenerating islet-derived family, member four [32]. Moreover, miR-24 also has a role in regulating cardiomyocyte apoptosis [33], smooth muscle cell proliferation [34], and phagocytosis by myeloid inflammatory cells [35]. Several reports have also revealed a critical role of miR-24 in cell differentiation. It has been recently reported that miR-24 and miR-27a are suppressors of embryonic stem cell differentiation [36]. Philipot *et al.* reported that miR-24 mediated chondrocyte terminal differentiation in osteoarthritis [37]. Overexpression of miR-24 suppresses fibrosis and the differentiation and migration of cardiac fibroblasts by regulating furin expression after myocardial infarction [38]. During adipocyte differentiation, miR-24 was found to be extensively downregulated, which regulated adipocyte differentiation by targeting fatty acid-binding protein 4 [39]. Interestingly, miR-24 was also found to be differentially expressed during mesenchymal stem cell differentiation toward osteoblasts [40]. However, the precise role and the underlying mechanism of miR-24 in regulating osteogenic differentiation have remained unexplored. Here, we demonstrate that miR-24 negatively regulates osteogenic differentiation by targeting Tcf-1. 

The direct interaction between miR-24 and Tcf-1 was detected by a dual luciferase reporter assay, which was further validated in a gain- or loss-of-function study in BMSCs and MC3T3-E1 cells. Tcf-1 is a critical target gene of the Wnt/β-catenin signaling pathway [18,19]. In recent years, targeting the Wnt/β-catenin signaling pathway to improve osteogenic differentiation has been widely studied. Overexpression of miR-346 activates Wnt/β-catenin and increases downstream gene expression by targeting Tcf-1 and inhibiting glycogen synthase kinase-3β, which promotes osteogenic differentiation [41]. Hassan *et al.* reported that miR-218 promoted the differentiation of bone marrow stromal cells by activating a positive Wnt signaling loop [42]. By targeting and inhibiting dickkopf-related protein 1, an antagonist of Wnt signaling, miR-335-5p was revealed to enhance bone formation and regeneration [43]. Similarly, miR-27 inhibits the gene expression of adenomatous polyposis coli leading β-catenin accumulation and thus Wnt activation to promote osteoblast differentiation [44]. Inhibiting low-density lipoprotein receptor-related protein 6 by miR-30e overexpression significantly downregulates β-catenin/Tcf transcriptional activity and dramatically inhibits osteoblast differentiation [45]. These reports indicate that targeting Wnt signaling by miRNAs has the potential to promote bone formation.

As a critical downstream gene of Wnt/β-catenin, Tcf-1 also plays an important role in regulating cell differentiation. Tcf-1 defective mice show decreased T cell differentiation [46]. Furthermore, a lack of Tcf-1 facilitates bone resorption in skeletal metabolism [47]. Here, we demonstrated that miR-24 regulates osteoblast differentiation possibly through regulating Tcf-1. It has reported that Runx2 is a target gene of β-catenin/Tcf-1 as overexpression of Tcf-1 increases Runx2 promoter activity and Runx2 gene expression in mouse pluripotent mesenchymal and osteo-progenitor cells [22]. In the present study, we have demonstrated that the expression levels of Tcf-1 and Runx2 are regulated by miR-24 in BMSCs and MC3T3-E1 cells. We further demonstrated that the silencing of Tcf-1 expression significantly abolished the positive effect of miR-24 inhibition on osteoblast differentiation.

## 4. Experimental Section

### 4.1. Cell Cultures

The murine osteoprogenitor cell line MC3T3-E1 and mouse bone mesenchymal stem cells (BMSCs) were purchased from Type Culture Collection of the Chinese Academy of Sciences (Shanghai, China). The cells were cultured in an α-modification of Eagle’s minimum essential medium (α-MEM; Life Technologies, Carlsbad, CA, USA) supplemented with 10% fetal bovine serum (FBS; Gibco, Los Angeles, CA, USA) and 1% penicillin/streptomycin in a humidified atmosphere containing 5% CO_2_ at 37 °C. For the induction of osteoblastic differentiation, BMSCs and MC3T3-E1 cells were grown in osteogenic differentiation medium (HyClone, Logan, UT, USA) containing 10% FBS supplemented with 10 mM sodium β-glycerophosphate, 50 μg/mL ascorbic acid, 10 nM dexamethasone, and 2 mM l-glutamine. The induction medium was renewed every two days for osteogenic differentiation.

### 4.2. Real-Time Quantitative Polymerase Chain Reaction (RT-qPCR)

For mRNA analysis, total RNA was extracted using TRIzol (Life Technologies, Carlsbad, CA, USA) that was then used to synthesize cDNA using M-MLV reverse transcriptase (Clontech, Palo Alto, CA, USA) according to the protocol of the manufacturer. For miRNA analysis, total RNA was extracted using the miRNeasy Mini Kit (Qiagen, Dusseldorf, Germany), which was then used to generate cDNA using the one-step primescript miRNA cDNA synthesis kit (Takara, Dalian, China) according to the manufacturer’s instructions. The quantification of gene expression levels was determined using SYBR Green qPCR Master Mix (ThermoFisher, Shanghai, China). The relative expression level was compared with an internal reference gene, *i.e.*, GAPDH (for mRNA) or U6 SnRNA (for miRNAs) using the 2^−ΔΔ*C*t^ method.

### 4.3. Alkaline Phosphatase (ALP) Activity

ALP activity was quantified by using ALP assay kit (Nanjing Jiancheng Bioengineering Institute, Nanjing, China) according to the instructions of the manufacturer. Briefly, cells were treated with 20 nM miR-24 precursor (miR-24) or miR-24 inhibitor (anti-miR-24) for 48 h and then cultured in differentiation medium for the induction of osteogenic differentiation for 3, 7 and 9 days. At the indicated time points, cells were harvested and washed with phosphate buffered solution (PBS) and lysed with lysis buffer followed by centrifugation (2500× *g* for 15 min at 4 °C). The supernatants were collected and incubated with SensoLyte *p*-nitrophenylphosphate. The absorbance at 405 nm was measured by a microtiter plate reader (ThermoElectron Corporation, Vantaa, Finland).

### 4.4. Measurement of Matrix Mineralization

Matrix mineralization was detected by Alizarin red S staining. Briefly, the treated cells were fixed with 70% ethanol for 1 h. After washing with distilled PBS, cells were stained with 40 mM Alizarin red S solution for 10 min followed by washing with distilled PBS to remove excessive stain. Alizarin red S stained mineral deposits were extracted and dissolved in 0.1 N NaOH. The absorbance at 540 nm was measured by a microtiter plate reader (ThermoElectron Corporation).

### 4.5. Western Blot Analysis

Proteins from cells were extracted using a protein extraction kit (Applygen Technologies, Beijing, China). The protein concentrations in different samples were measured using the Bio-Rad protein assay kit (Bio-Rad, Hercules, CA, USA). For protein isolation, an equal amount (25 μg) of protein from each sample was separated by 12.5% sodium dodecyl sulfate polyacrylamide gel electrophoresis (SDS-PAGE) and then then transferred to a nitrocellulose membrane (Bio-Rad). The membranes were blocked with 3.0% nonfat milk for 1 h at 37 °C, then incubated with primary antibodies at 4 °C overnight. Thereafter, horseradish peroxidase conjugated secondary antibodies (1:2000; Bioss, Beijing, China) were added and incubated with for 1 h at room temperature. The immune-reactive protein bands on the membrane were detected using an enhanced chemiluminescence detection system (Amersham, Little Chalfont, UK). The primary antibodies used in these experiments, including anti-Tcf-1 and anti-GAPDH antibodies, were purchased from Santa Cruz Biotechnology (Santa Cruz, CA, USA).

### 4.6. Dual Luciferase Reporter Assay

The cDNA fragment of 3'-UTR of Tcf-1 containing the putative binding sites of miR-24 was amplified and then subcloned into pGL3 luciferase promoter vector (Promega, Madison, WI, USA). A total of 0.5 μg pGL3-Tcf-1 3'-UTR plasmids were co-transfected with 20 nM miR-24 or the miR-24 inhibitor into MC3T3-E1 cells using Lipofectamine 2000 transfection reagent (Invitrogen, Carlsbad, CA, USA) according to the manufacturer’s protocol. Cells were harvested after 48 h of transfection and incubation, and the relative luciferase activity was measured using a dual luciferase reporter assay kit (Promega).

### 4.7. Small Interfering RNA (siRNA) Transfection

Cells were grown in serum-free medium for 1 h before siRNA transfection was performed. The cells were transfected with 50 nM of Tcf-1 siRNA and control siRNA (Santa Cruz Biotechnology, Santa Cruz, CA, USA) using Lipofectamine 2000 and incubated for 10 h. After that, cells were subjected to the osteogenic induction protocol.

### 4.8. Data Analysis

Data are represented as means ± standard deviation (SD). Statistical differences were analyzed by SPSS version 11.5 (SPSS Inc., Chicago, IL, USA) using one-way ANOVA followed by Bonferroni post hoc. Differences were considered statistically significant with *p*-values less than 0.05.

## 5. Conclusions

In conclusion, our data indicate that miR-24 acts as an important regulator of osteogenic differentiation by targeting and regulating Tcf-1. Our findings provide novel insight into miR-24, which may serve as an effective target in bone formation and regeneration. However, further studies are warranted to validate the exact role of miR-24 in animal models *in vivo*.
